# A Comparison of Treatment-Seeking Behavioral Addiction Patients with and without Parkinson’s Disease

**DOI:** 10.3389/fpsyt.2017.00214

**Published:** 2017-11-03

**Authors:** Anne Sauvaget, Susana Jiménez-Murcia, Fernando Fernández-Aranda, Roser Granero, Marie Grall-Bronnec, Caroline Victorri-Vigneau, Samuel Bulteau, Pascal Derkinderen, Jean M. Vanelle, Anders Hakansson, Gemma Mestre-Bach, Trevor Steward, José M. Menchón

**Affiliations:** ^1^Department of Psychiatry, Institut d’Investigació Biomédica de Bellvitge (IDIBELL), Bellvitge University Hospital, Barcelona, Spain; ^2^Addictology and Liaison Psychiatry Department, Nantes University Hospital, Nantes, France; ^3^CIBER Fisiopatología Obesidad y Nutrición (CIBEROBN), Instituto de Salud Carlos III, Madrid, Spain; ^4^Department of Clinical Sciences, School of Medicine, University of Barcelona, Barcelona, Spain; ^5^Departament de Psicobiologia i Metodologia de les Ciències de la Salut, Universitat Autònoma de Barcelona, Barcelona, Spain; ^6^INSERM, SPHERE U1246, University of Nantes, Nantes, France; ^7^INSERM, SPHERE U1246, University of Tours, Nantes, France; ^8^Clinical Pharmacology Department, Centre for Evaluation and Information on Pharmacodependence, Nantes University Hospital, Nantes, France; ^9^INSERM, U913, Nantes, France; ^10^Department of Neurology, CHU Nantes, Nantes, France; ^11^Faculty of Medicine, Department of Clinical Sciences Lund, Psychiatry, Lund University, Lund, Sweden; ^12^CIBER Salud Mental (CIBERSAM), Instituto de Salud Carlos III, Madrid, Spain

**Keywords:** Parkinson’s disease, personality, impulsivity, behavioral addictions, gambling disorder, impulse control disorders

## Abstract

The administration of dopaminergic medication to treat the symptoms of Parkinson’s disease (PD) is associated with addictive behaviors and impulse control disorders. Little is known, however, on how PD patients differ from other patients seeking treatments for behavioral addictions. The aim of this study was to compare the characteristics of behavioral addiction patients with and without PD. *N* = 2,460 treatment-seeking men diagnosed with a behavioral addiction were recruited from a university hospital. Sociodemographic, impulsivity [Barratt Impulsiveness Scale (BIS-11)], and personality [Temperament and Character Inventory-Revised (TCI-R)] measures were taken upon admission to outpatient treatment. Patients in the PD group were older and had a higher prevalence of mood disorders than patients without PD. In terms of personality characteristics and impulsivity traits, PD patients appeared to present a more functional profile than PD-free patients with a behavioral addiction. Our results suggest that PD patients with a behavioral addiction could be more difficult to detect than their PD-free counterparts in behavioral addiction clinical setting due to their reduced levels of impulsivity and more standard personality traits. As a whole, this suggests that PD patients with a behavioral addiction may have different needs from PD-free behavioral addiction patients and that they could potentially benefit from targeted interventions.

## Introduction

Researchers and clinicians have identified that the administration of dopaminergic medication to ameliorate the symptoms of Parkinson’s disease (PD) is associated with impulse control disorders (ICDs) (1). These disorders vary greatly in their symptomatology and include the behavioral addictions: gambling disorder, compulsive buying, and hypersexuality. Although prevalence studies are scarce, ICDs are thought to affect up to 16% of PD patients (2). Due to their clinical similarities with substance abuse disorders, many ICDs have been reclassified as behavioral addictions; and in the latest version of the Diagnostic and Statistical Manual of Mental Disorders (DSM-5), gambling disorder was moved from the category of ICD to the new category of “Substance-Related and Addictive Disorders” (3).

The majority of studies examining PD patients with behavioral addictions have been cross-sectional, thereby greatly limiting the ability to reach meaningful conclusions regarding causal factors. One recent longitudinal study examining PD patients with an ICD over a 2-year span found that the prevalence of ICD behaviors remained relatively stable across observational period and that ICD-positive patients had more severe depression and reduced quality of life (4). Studies have identified that PD patients who develop behavioral addictions tend to be younger, more impulsive, and are more likely to have a personal or family history of alcohol abuse than PD patients without behavioral addictions (5, 6). High levels of impulsivity, which is understood to be modulated by dopaminergic transmission in the ventral striatum, may represent a risk factor for the development of behavioral addictions (7). One study comparing patients with PD to healthy controls found that both male gender and higher impulsivity scores, but not dose and kind of dopaminergic medications, were associated in PD with an increased probability of ICDs (8). Likewise, another study examining impulsivity traits in drug-naïve patients found that elevated levels of attentional impulsiveness scores in PD patients screening positive for ICDs, leading the authors to suggest that these patients may possesses a subclinical pattern characterized by a reduced ability to focus on the task at hand (9). Contrastingly, the majority of studies in medicated PD patients report that, overall, PD patients tend to have lower levels of impulsivity compared to healthy controls (10). Significantly higher incidence of anhedonia and higher levels of impulsivity traits have also been found to coincide in patients with PD and an ICD, meaning that impairment of hedonic capacity coupled with higher impulsivity levels possibly might facilitate loss of control over reward-related behavior, thereby favoring the shift toward predominantly habit-based compulsive behaviors (11). Furthermore, both depression and anxiety symptoms have been found to be significantly correlated to impulsivity scores on the Barratt Impulsiveness Scale (BIS-11), indicating that PD patients with ICDs may be at higher risk for mood disorders (12).

Indeed, some authors have used the term “premorbid personality” to characterize distinctive features of PD patients’ personality traits, which are defined by a diminished desire to explore new environmental stimuli (“novelty seeking”) and higher levels of harm avoidance (10). Therefore, these patients would likely show a tendency toward inflexibility, low impulsivity levels, introspection, and cautiousness, and all this traits would remain after the manifestation of PD motor symptoms (13, 14). The few studies examining personality traits of PD patients with behavioral addictions have found that these patients are more likely to show a tendency to lie and to have higher scores on cynicism and bizarre ideation (15), as well as lower levels of agreeableness and conscientiousness (16).

Addictive behaviors in PD patients are frequently under-reported by both patients and caregivers (17). This fact poses a challenge for clinicians treating Parkinson’s patients with a behavioral addiction. To our knowledge, no studies to date have compared PD patients with behavioral addictions to PD-free patients with behavioral addictions. Clarifying the extent to which differences between these populations exist is needed to determine whether customized addiction treatment interventions are needed for PD patients. As such, the aim of the present study was to compare sociodemographic, personality, and impulsivity traits in patients seeking treatment for behavioral addictions with and without PD. By comparing behavioral addiction patients with and without PD, our findings could help make evident for behavioral addiction clinicians the key characteristics that distinguish one group from another.

## Materials and Methods

### Sample

A sample of 2,460 men diagnosed with a behavioral addiction from the Department of Psychiatry at Bellvitge University Hospital (Barcelona, Spain) between February 2004 and November 2014 was considered. Exclusion criteria for the study were the presence of a mental disorder, intellectual disability, or active psychotic disorder. Only men were included in our analyses due to the lack of women seeking treatment at our Unit. Patients were screened *via* a structured interview by experienced clinical psychologists and psychiatrists before being included in the study sample. The sample of *N* = 2,460 patients was classified into two groups according to the presence/absence of PD (*n* = 32 with PD and *n* = 2,428 without PD).

### Measures

We evaluated the presence of behavioral addictions with the following tools: patients were diagnosed with pathological gambling if they met DSM-IV-TR criteria (18). All patient diagnoses were reassessed and recodified *post hoc* and only patients who met DSM-5 criteria for GD were included in our analysis (3). Compulsive buying was assessed according to McElroy et al.’s criteria (19), sexual addiction according to the sexual disorders not otherwise specified section (302.70) of the DSM-5 (3), and Internet gaming disorder according to Griffiths and Hunt (20) and DSM-5 (3) criteria.

Personality was assessed using the Temperament and Character Inventory-Revised (TCI-R) (21). This questionnaire is structured on seven primary dimension scores: four temperamental factors and three character dimensions. These different personality dimensions have demonstrated adequate reliability-validity in the Spanish population with Cronbach’s alphas between 0.77 and 0.84 (22).

Lastly, impulsivity was measured using the BIS-11 (23). This includes 30 items rated on a four-point Likert scale. The validated Spanish version of this instrument (24) was used in this study.

### Additional Data

Demographic, clinical, and social/family variables were measured using a semi-structured, face-to-face clinical interview (25). Some of the variables covered were behavioral addiction duration, current pharmacological treatment, and psychiatric comorbidities.

### Procedure

Parkinson’s disease patients were first diagnosed by a neurologist and then derived to the Department of Psychiatry at our University Hospital in order to receive evaluation regarding behavioral addiction options. Patients individually completed the questionnaires required for this study before initiating outpatient treatment. If patients were unable to complete the evaluation on their own (e.g., due to being illiterate), these instruments were administered by a clinician. Somatic comorbidities and psychopharmacological treatments were assessed, especially in the case of dopamine replacement therapy and psychotropic drugs.

### Statistical Analysis

Statistical analysis was carried out with SPSS20 for windows. Chi-square (χ^2^) tests compared categorical variables between patients with and without PD. Analysis of variance (ANOVA) adjusted for patients’ age and behavioral addiction subtype compared the quantitative clinical variables. The effect size for the proportions and means comparisons was estimated through Cohen’s-*d* coefficient, considering *|d|* > 0.50 to be a moderate effect size and *|d|* > 0.80 to be a high effective size. Bonferroni–Finner’s correction was applied to control Type-I error due to multiple statistical comparison.

## Results

### Descriptive for the Sample

Table 1 displays a description of the study sample and the groups with and without PD. Significant differences were found between participants with and without PD with regards to age, employment status, and civil status (patients in the PD group were older and had a higher proportion of married subjects). Table S1 in Supplementary Material contains the prevalence of other behavioral addictions in the sample. Table S2 in Supplementary Material lists the medication being taken by the *n* = 32 patients diagnosed with PD. The duration of PD for this group was 7.7 years (SD = 5.5).

**Table 1:** Description of the sample.

		Total *n* = 2,460		Parkinson = absent *n* = 2,428		Parkinson = present *n* = 32		*p*
Age (years old)	*Mean-SD*	41.8	13.5	41.5	13.4	61.7	6.94	<0.001*
Origin; *n %*	*Spanish*	2,282	92.8%	2,250	92.7%	32	100.0%	0.112
Education level; *n %*	*Primary*	1,866	75.9%	1,846	76.0%	20	62.5%	0.109
	*Secondary*	444	18.0%	437	18.0%	7	21.9%	
	*University*	150	6.1%	145	6.0%	5	15.6%	
Employment; *n %*	*Employed*	1,420	57.7%	1,415	58.3%	5	15.6%	<0.001*
Civil status; n %	*Single*	824	33.5%	822	33.9%	2	6.3%	0.004*
	Married	1,320	53.7%	1,296	53.4%	24	75.0%	
	*Divorced*	316	12.8%	310	12.8%	6	18.8%	

*Bold: significant comparison (0.05 level).

### Comparison of Patients with and without PD

Table 2 contains a comparison of the clinical variables between the patients with and without PD. Adjusted for the covariates age and behavioral addiction subtype, the presence of PD was associated with a lower prevalence of substances use/abuse (tobacco, alcohol, and other drugs), lower levels in impulsivity (except for on the BIS-11 motor scale) and higher levels in the personality traits of reward dependence, self-directedness, and cooperativeness. Patients with PD also presented a higher prevalence of mood disorders than patients without PD.

**Table 2:** Comparison between patients with and without Parkinson disorder.

	Parkinson = No (*n* = 2,428)		Parkinson = Yes (*n* = 32)		Comparison between groups		
*Psychiatric commorbidities^a^*	*n*	%	*n*	%	χ^2^(1)	*p*	|*d*|
Family history of mental disorders	1,361	56.1%	16	50.0%	0.47	0.493	0.12
History of psychiatric problems	1,000	41.2%	17	53.1%	1.86	0.173	0.24
Psychiatric disorders at present	731	30.1%	10	31.3%	0.02	0.889	0.02
Mood disorder	246	10.1%	7	21.9%	4.72	0.030*	0.32
Anxiety disorder	153	6.3%	2	6.3%	0.00	0.990	0.00
Alcohol abuse	369	15.2%	0	0%	5.72	0.017*	**0.60^†^**
Substance abuse	247	10.2%	0	0%	6.82	0.009*	**0.52^†^**

*Addiction-related measures^b^*	Mean	SD	Mean	SD	*F_(1; 2,445)_*	*p*	*|d|*
Age of addiction onset (years old)	28.06	11.37	46.95	12.39	87.05	0.001*	**1.59^†^**
Addiction duration (years)	13.38	7.76	13.95	9.85	0.17	0.683	0.06
*Barratt Impulsiveness Scale-11 scales*^b^							
Attentional	16.01	2.87	11.92	3.39	59.39	0.001*	**1.30^†^**
Motor	18.07	4.57	17.51	4.52	0.45	0.505	0.12
Non-planning	24.03	4.22	20.14	3.33	25.15	0.001*	**1.02^†^**
Total score	58.15	9.20	49.56	8.67	25.71	0.001*	**0.96^†^**
*Temperament and Character Inventory-Revised scales^b^*							
Novelty seeking	108.67	13.49	106.71	7.73	0.67	0.413	0.18
Harm avoidance	100.44	15.64	98.71	11.77	0.37	0.544	0.13
Reward dependence	99.21	14.38	105.53	10.12	5.74	0.017*	**0.53^†^**
Persistence	109.88	19.76	109.60	15.59	0.01	0.936	0.02
Self-directedness	128.43	20.13	136.41	17.20	4.68	0.031*	**0.50^†^**
Cooperativeness	131.84	16.27	138.11	11.62	4.43	0.035*	**0.51^†^**
Self-transcendence	63.93	14.15	63.89	9.42	0.00	0.987	0.00

*^a^Results obtained in χ^2^ tests*.

*^b^Results obtained in analysis of variance*.

*|d|: Cohen’s-d coefficient*.

*Results adjusted by the participants’ age and behavioral addiction subtype*.

*Bold: significant comparison (0.05 level).

*^†^Bold: moderate (|*d*| > 0.50) to high (|*d*| > 0.80) effect size*.

*p-Value includes Bonferroni–Finner’s correction for multiple statistical comparisons*.

## Discussion

This study analyzed the specific sociodemographic, impulsivity, and personality features of PD patients with addictions compared to PD-free patients with addictions. It was our aim to provide behavioral addiction clinicians with a clearer picture of the features that distinguish these two groups from each other. Regarding sociodemographic variables, age greatly differed between both groups, with older ages being found in the PD group. This finding dovetails with several studies reporting that the PD is most often found in men over 60 years of age (26). Moreover, our results are consistent with other studies supporting a higher prevalence of mood disorders in this population (27, 28). Some studies suggest that approximately 30–40% of PD patients undergo depression, evidencing a strong association between both disorders (29). Mood disorders are, in fact, one of the most characteristic non-motor manifestations of PD and they could be experienced at stage of the disease (30, 31).

Some discrepant results emerged when examining the psychological profile of PD patients. On the one hand, previous studies have reported higher novelty-seeking trait scores (understood as a greater tendency to becoming excited about unfamiliar contexts) in PD patients with comorbid behavioral addictions, mainly gambling disorder, than in PD patients without behavioral disorders (16, 32, 33). However, the significant differences found between the two groups in personality dimensions support the position that treatment-seeking patients with PD present a more functional personality profile than their PD-free counterparts. However, contrary to expectations, this study did not find significant differences between both groups in this personality dimension. On the other hand, taking the other personality dimensions into account, the significant differences found between the two groups support the position that treatment-seeking patients with PD present a more functional personality profile than their PD-free counterparts. Higher scores in reward dependence and cooperativeness would indicate that these patients are tolerant and socially empathetic, despite the interference that PD motor symptomatology may cause in their social interactions. In turn, higher self-directedness levels would allow them to have clearer life goals and adaptively orient their behavior toward them (34). These dimensions would facilitate, among other aspects, greater adherence to treatment.

Moreover, evident differences in impulsivity traits appeared when comparing both groups: the PD group with a behavioral addiction showed considerably lower attentional and non-planning impulsivity compared to PD-free patients with a behavioral addiction. Both these traits reflect an inability to wait for a reward and a rapid response style without proper premeditation, traits that are commonly seen in behavioral addictions such as gambling disorder (35). This may appear to be a stark contrast to previous studies reporting that PD patients with an ICD present higher levels of impulsivity compared to other PD patients; however, it must be noted that behavioral addiction patients are often sociodemographically very distinct from PD patients (8). Therefore, our findings suggest that PD patients with a behavioral addiction may be more difficult to identify in clinical practice, especially during the initial stages of PD. As advocated for by other researchers, careful investigation of risk behaviors is needed in routine clinical settings, as these behaviors are rarely spontaneously reported to neurologists (9).

It is especially worth highlighting that our PD patient sample with a behavioral addiction presented lower levels of substance and alcohol use/abuse compared to the PD-free group. In the case of gambling disorder, for example, one meta-analysis found that the prevalence of a substance use disorder in gamblers was 57.5% (36). Since PD patients are not widely thought to be at increased risk of substance abuse, this raises the likelihood that behavioral addictions in PD patients may risk going undetected if they are not properly screened (37). Future research should focus on the development of new treatment interventions taking this phenotype into account.

### Limitations

The findings of this study should be considered with certain drawbacks in mind. First, our study did not examine PD severity. Relatedly, cognition impairment was not assessed, and it has been postulated that PD patients with behavioral addictions have more severe frontal lobe dysfunction than PD patients without behavioral addictions (38, 39). Second, possibly due to higher awareness of this condition, the number of GD patients in our sample was vastly higher than the number of other behavioral addiction patients. Future research should include larger, more balanced samples so as to overcome these limitations. In this same vein, all data were collected from men who sought treatment and it significantly limits the generalizability of the present study results to women and other populations. Moreover, another issue that was not addressed in this study was whether the onset of the behavioral addiction occurred prior to- or following receiving PD diagnosis. Lastly, it would have been of interest to include a PD control group and larger sample of PD and behavioral addiction patients.

## Ethics Statement

The present study was carried out in accordance with the latest version of the Declaration of Helsinki. The University Hospital of Bellvitge Ethics Committee of Clinical Research approved the study, and signed informed consent was obtained from all participants.

## Author Contributions

Research project elaboration: AS, SJ-M, FF-A, and JMM; Organization: AS, GM-B, TS, and SJ-M; Execution: AS, RG, MG-B, CV-V, SB, PD, JMV, AH, GM-B, and TS; Design: SJ-M, AS, FF-A, TS, GM-B, and RG; Statistics execution: RG; Review and Critique: AS, MG-B, GM-B, TS; SJ-M, RG; Writing of the first draft: AS; Review and Critique: AS, MG-B, CV-V, SB, JMM, RG, GM-B, TS.

## Conflict of Interest Statement

The authors declare that the research was conducted in the absence of any commercial or financial relationships that could be construed as a potential conflict of interest.
