# Supplementary material for: A Comparison of Treatment-Seeking Behavioral Addiction Patients with and without Parkinson’s Disease
Source: Front Psychiatry. 2017 Nov 3;8:214. doi: 10.3389/fpsyt.2017.00214 (PMC5675875; doi:10.3389/fpsyt.2017.00214)
Supplement: Supplementary file 1 [file table_1.doc]

*Table S1 (supplementary)*

Distribution of the behavioral addictions and comparison between groups

|  | Parkinson=No  (*n*=2,428) | | Parkinson =Yes  (*n*=32) | |  | | |
| --- | --- | --- | --- | --- | --- | --- | --- |
|  | *n* | *%* | *n* | *%* | χ^2^ *(df=1)* | *p* | *|d|* |
| Number behavioral addictions |  |  |  |  |  |  |  |
| One | 2414 | 99.4% | 30 | 93.8% | 15.73 | **<.001** | 0.32 |
| Two | 14 | 0.6% | 2 | 6.3% |  |  |  |
| Gambling disorder | 2346 | 96.6% | 27 | 84.4% | 13.89 | **<.001** | 0.43 |
| Slot machines | 2125 | 87.5% | 27 | 84.4% | 0.29 | .593 | 0.09 |
| Bingo | 1153 | 47.5% | 11 | 34.4% | 2.18 | .140 | 0.27 |
| Lotteries | 2006 | 82.6% | 22 | 68.8% | 3.29 | .070 | 0.33 |
| Casino | 561 | 23.1% | 8 | 25.0% | 0.06 | .801 | 0.04 |
| Cards | 910 | 37.5% | 8 | 25.0% | 2.10 | .147 | 0.27 |
| Horse races | 78 | 3.2% | 0 | 0.0% | 1.06 | .303 | 0.26 |
| Sports | 304 | 12.5% | 1 | 3.1% | 2.57 | .109 | 0.36 |
| Other behavioral addiction subtypes |  |  |  |  |  |  |  |
| Compulsive buying behavior | 35 | 1.4% | 2 | 6.3% | 2.22 | .136 | 0.25 |
| Sex addiction | 21 | 0.9% | 2 | 6.3% | 9.89 | **.002** | 0.29 |
| Videogames addiction | 35 | 1.4% | 1 | 3.1% | 0.62 | .431 | 0.11 |
| Other | 2 | 0.1% | 1 | 3.1% | 24.01 | <.001 | 0.24 |

*Note.* *Bold: significant comparison (.05 level).

*Table S2 (supplementary)*

Medication usage of the Parkinson’s disease (n=32).

|  | *n* | *%* |
| --- | --- | --- |
| Levodopa | 22 | 68.8% |
| Catechol-O-methyltransferase inhibitors | 15 | 46.9% |
| Dopamine agonists | 20 | 62.5% |
| Monoamine oxidase inhibitors | 10 | 31.3% |
| Others | 4 | 12.5% |
| Anticholinergic drugs | 0 | 0% |
| Antidepressants | 10 | 31.3% |
| Anxiolytics | 10 | 31.3% |
| Antipsychotics | 5 | 15.6% |
| Mood stabilizers | 1 | 3.1% |
| Hypnotics | 2 | 6.3% |
